# Smart and Regenerative Urban Growth: A Literature Network Analysis

**DOI:** 10.3390/ijerph17072463

**Published:** 2020-04-03

**Authors:** Angeliki Peponi, Paulo Morgado

**Affiliations:** 1 Faculty of Environmental Sciences, Czech University of Life Sciences Prague, Kamýcká 129, 16500 Praha—Suchdol, Czech Republic; 2 Institute of Geography and Spatial Planning, Centre of Geographical Studies, Universidade de Lisboa, Rua Branca Edmée Marques, 1600-276 Lisboa, Portugal; paulo@campus.ul.pt

**Keywords:** bibliometric network, distance maps, smart and regenerative urban growth, urban ecology, urban metabolism

## Abstract

“Smart city”, “sustainable city”, “ubiquitous city”, “smart sustainable city”, “eco-city”, “regenerative city” are fuzzy concepts; they are established to mitigate the negative impact on urban growth while achieving economic, social, and environmental sustainability. This study presents the result of the literature network analysis exploring the state of the art in the concepts of smart and regenerative urban growth under urban metabolism framework. Heat-maps of impact citations, cutting-edge research on the topic, tip-top ideas, concepts, and theories are highlighted and revealed through VOSviewer bibliometrics based on a selection of 1686 documents acquired from Web of Science, for a timespan between 2010 and 2019. This study discloses that urban growth is a complex phenomenon that covers social, economic, and environmental aspects, and the overlaps between them, leading to a diverse range of concepts on urban development. In regards to our concepts of interest, smart, and regenerative urban growth, we see that there is an absence of conceptual contiguity since both concepts have been approached on an individual basis. This fact unveils the need to adopt a more holistic and interdisciplinary approach to urban planning and design, integrating these concepts to improve the quality of life and public health in urban areas.

## 1. Introduction

Humankind alters the earth´s natural processes and shapes the landscapes causing alterations in global scale phenomena such as land use/land cover change, economy, energy, transport, population, and urbanization, among others [1]. Globally, cities expand, and their population is growing; one in five people on earth lives in a city with a population of more than one million, and sixty percent of the global population is projected to live in urban settlements by the year 2030 [2]. At the European level, we notice two extremes; around hundred sixty five million citizens live in dynamically growing cities mainly due to migration, and around twenty five million citizens live in “dynamically shrinking” cities [3]. Approximately forty percent of European cities with a population of more than two hundred thousand are witnessing urban shrinkage from economic and demographic perspectives [4]. Urban shrinkage is caused mainly due to changes in economic demographic and political systems as well as environmental hazards, and it leads to “under-utilization, vacancy, demolition, emerging brownfield sites, and de-densification of spaces” [3]. The analysis of the dynamics and the spatial configuration of the trends of urban growth consists of an essential topic in current urban studies [5].

Urban growth has a double meaning; on one hand, it signifies the constant rise of urban population (urbanization) and, on the other hand, the expansion of urban lifestyle and infrastructure within the settlement system [6,7]. Urban growth offers a variety of opportunities (economic, social, political growth), but it has a negative impact as well. Urban sprawl is the type of urban growth having a negative meaning [5,8]. Despite the dialogue about the definition of urban sprawl, it represents overall a wasteful type of urbanization. It is related to an uncontrolled expansion of urban areas, scattered settlement areas (how dense or scattered are the buildings and patches of built-up areas within the landscape), and low-density development (high area of land per person) [9,10,11]. Urban sprawl has significant negative impacts regarding land use/land cover change and energy efficiency, urban economy, social structure, physical environment, public health, as well as the form and spatial arrangement of urban development [12,13,14]. 

Although there is a growing body of empirical studies that analyses urban growth and reveals its impacts, less attention has been devoted to studies that review the evolution of various concepts on sustainable urban development. This paper seeks to explore the evolution of the emerging concepts on sustainable urban development (smart city, sustainability, regenerative city, and urban metabolism) through a novel network analysis of the existing literature, using VOSviewer software. 

Initially, we attempt to disclose the main research trends found in the literature under review. Then, we detect the key scholarly sources considering firstly, the number of their citations, and secondly, their overall conceptual relevance to the topic under review. Looking at the way that these key scholarly sources are connected, we reveal the sub-research trends. The next steps are to analyze the key concepts and theories coming from these key scholarly sources and find their origins and connections. The last step of the literature network analysis is to detect the most influential authors and see how they are related to each other.

VOS mapping and clustering techniques are both promising and useful. They have been applied to conduct bibliometric analysis in various fields of studies for instance co-occurrence term analysis in psychology [15], bibliographic analysis of the concept safety culture [16] of the Journal of Infection and Public Health [17], of thermal comfort and building control research [18] and a bibliometric analysis on connection between urban governance, planning, design and development [19] among others. Thus, we adopted the software and adapted the network analysis algorithms to decode the degree of connectivity between smart and regenerative urban growth concepts.

The remainder of this study is organized as follows. The Section 2 describes the methodology applied to conduct the literature network analysis. Initially, Section 2.1 provides information regarding the data acquisition; Section 2.2 presents the theoretical background and the technical settings for the construction of the desire bibliographic networks. In Section 3, the key findings of the literature network analysis are accompanied by maps and tables. The Section 4 discusses the methodology, results, and limitations of the literature network analysis review, and Section 5, the overall contribution of this paper to the field of urban and environmental planning. 

## 2. Review Method 

A methodology comprises a set of applied procedures and techniques, unveiling information regarding a specific topic or research subject, to provide overall scientific credibility of the study. Similarly, the literature review should have a specific and tailored methodology, instead of being opaque or even randomly and unstructured made as in most of the papers. Considering this, the complexity of the topic under study, and its societal significance, there is a need for a multidisciplinary systematic literature network analysis able to provide scientific evidence upon the conceptual evolution of sustainable urban growth.

**Figure 1:**
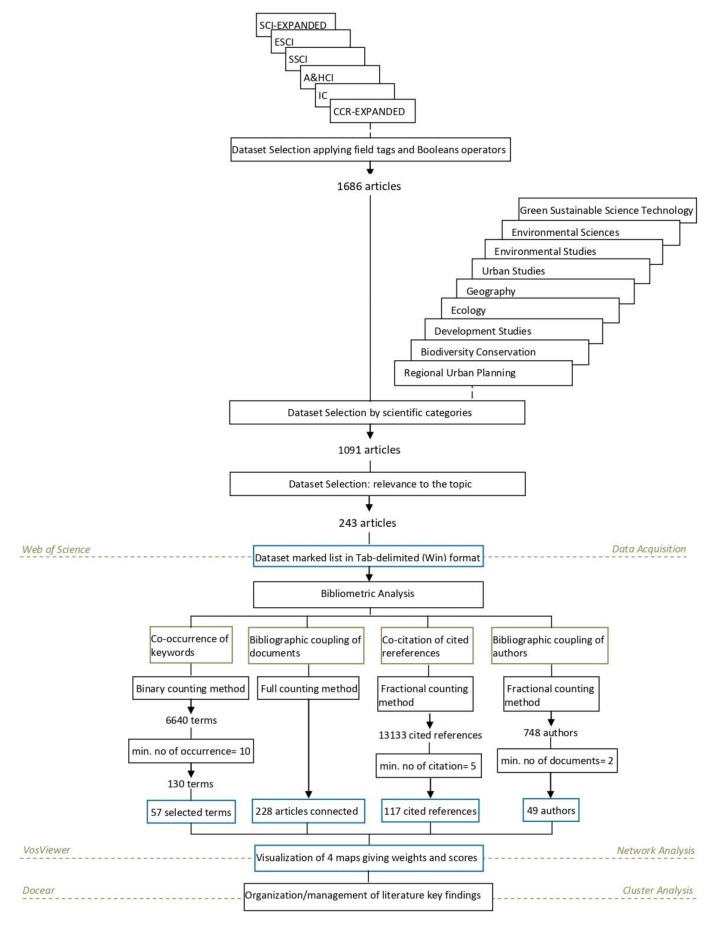
Flowchart of the developed methodology.

Here, we have conducted a literature network analysis using Web of Science (WOS) as the main bibliographic data source, VOSviewer software for the bibliometric network analysis and visualization. Docear software was used to organize and manage the key findings of the literature, and Mendeley software was used to generate the references and citations to scientifically support the idea of smart and regenerative redesign of urban areas (Figure 1).

### 2.1. Data Acquisition

To acquire the relevant literature, the Web of Science Core Collection database was selected, applying an advanced search filter by using field tags and Booleans operators as in the following expressions; (TI=(smart* AND urban) OR TI=(sustainable AND urban) OR TI=(regenerat* AND urban) OR TI=(metabolism) AND TS=(urban AND sprawl)), (TI= (urban AND metabolism)), where TI refers to the title of the document and TS to the topic. We used timespan between 2010 and 2019, English language, Article type of document for the search and Science Citation Index Expanded (SCI-EXPANDED), Social Sciences Citation Index (SSCI), Arts & Humanities Citation Index (A&HCI) Emerging Sources Citation Index (ESCI), Current Chemical Reactions (CCR-EXPANDED), Index Chemicus (IC) indexes.

These two searches resulted initially in 1686 related articles in total. We reduced the amount of literature to 1091 selecting specific WOS categories (Environmental Sciences OR Environmental Studies OR Urban Studies OR Green Sustainable Science Technology OR Regional Urban Planning OR Geography OR Ecology OR Development Studies OR Biodiversity Conservation). From this search set, we created a marked list selecting finally 243 articles considering their relevance to the topic and the times cited by reading their title, abstract and keywords. These 243 records were saved in Tab-delimited (Win) format considering their full records and cited references.

### 2.2. Bibliometric Analysis

To construct and analyze our bibliometric network of the 243 articles, we used VOSviewer software. VOS mapping technique is applied to create distance maps. In distance maps, the distance between two items of the network shows the strength of their relatedness; shorter distance means higher relatedness. This method comes as an alternative to the multidimensional scaling technique traditionally used for the visualization of these types of maps [20,21,22]. The VOS mapping technique consists of three parts; a) the normalization, b) the mapping, and c) the clustering of the network nodes. In the first part, the association strength normalization is performed by default, normalizing the strength/weight of the links between the items of the nodes. The second part is the two-dimensional mapping of the nodes of the bibliometric network placing the nodes with strong relation closer to each other and the nodes with weak relation in longer distance to each other. In the third part, the clustering technique is applied, which assigns each node of the network to clusters considering their relatedness. More information regarding the expressions applied from the VOS mapping technique can be found in [23,24,25].

Depending on the type of analysis we want to conduct, the items of our interest can be connected by co-authorship, co-occurrence, citation, bibliographic coupling, or co-citation links calculated in one of two ways. The full counting versus the fractional counting method is used to calculate bibliographic coupling, co-citation, or co-authorship links and the binary versus the full counting method is used to calculate co-occurrence links in networks/ maps created based on text data.

**Figure 2:**
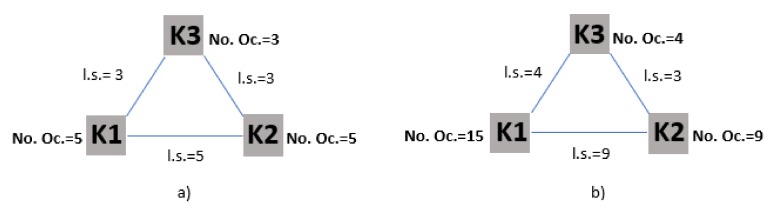
Co-occurrence network of keywords, (**a**) binary counting method, (**b**) full counting method.

**Figure 3:**
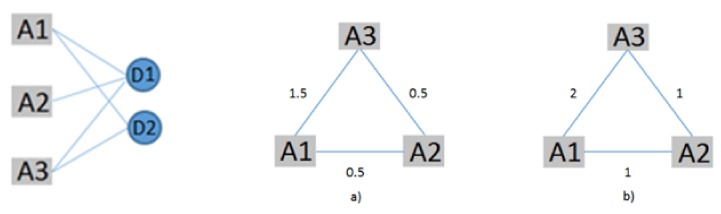
Bibliographic coupling network of authors, (**a**) fractional counting method, (**b**) full counting method.

Initially, we created a co-occurrence map based on text data to see which keywords/terms are related to each other in our bibliographic data set, revealing the research trends in our data set. The terms were extracted from both the title and abstract fields of the documents. For the construction of this map/network, we used binary counting method instead of full counting (Table 1, Figure 2). In this way, the co-occurrence links between the keywords are based on the number of documents that they occur together at least once. Looking at Table 1, we see the number of occurrences of three keywords in five documents. Figure 3, demonstrates the number of occurrences (No Oc.) and the strength of the links (l. s.) between the keywords using binary and full counting method. Applying the full counting method signifies that all occurrences of a term in all documents are counted. On the contrary, using binary counting method, the number of occurrences of a term is not taken into consideration; only the presence or the absence of a term in a document counts. Defining a minimum number of occurrences of a term equaling ten, from 6640 terms 130 meet the threshold, and 57 were selected as the most relevant terms based on our interpretation and their relevance score. Terms with higher relevance scores tend to represent specific concepts under study, while terms with lower relevance score appear to represent more general topics. The network was normalized with the association strength method and clustered with resolution parameter γ equals one and the minimum cluster size equals five. The co-occurrence links were weighted considering the occurrence of the terms. The average of citations was used as the score attribute.

Then, we constructed a bibliographic coupling network of documents intending to see which are more related to each other and more cited. In this map, the relatedness of the documents is based on the degree that they cite the same document. Considering that all the documents of our bibliometric network are related to the main topic, we used the full counting method to highlight the influence of high cited documents to the network. From the total 243 documents, the 228 were connected. We mapped and visualized the network into six clusters using association strength as a normalization method with resolution parameter equaling one and minimum cluster size equaling five. The bibliographic coupling links were weighted using the total link strength, and the number of citations was used as the score attribute.

Afterwards, we constructed the bibliographic coupling network of authors using the fractional counting method to examine which authors share a common field of studies. Giving the minimum number two for documents per author, of the 748 authors, only 49 meet this threshold. In this network, the relatedness of the authors is based on the degree that they cite the same document. In this way, the bibliographic coupling links between the authors are based on the number of documents that they commonly cite, not including the total number of authors of each of the same documents that they cite. For example, if an author A2 cites the same document D1 with the authors A1 and A3, the links between the author A2 and A1 and A2 and A3 will have strength of 1 / 2= 0.5, and at the same time if the authors A1 and A3 have cited another document D2, the strength of the link between A1 and A3 will be 1.5 (1/2 =0.5 for the D1, plus 1 for the D2) (Figure 3). We mapped the network into four clusters using association strength as a normalization method. The minimum cluster size was equal to five. We gave the total link strength the same score as the weight and the average of citations as the attribute of the items. 

Thereafter, we intended to see the relatedness of the cited references based on the degree that they have been cited together by another document. For this type of analysis, we constructed a co-citation map of cited references using the fractional counting method to avoid the influence of documents with a long list of references, as mentioned previously. Giving a minimum number of citations of a cited reference equals to five from 13133 cited references 117 meet this threshold.

## 3. Results

**Figure 4:**
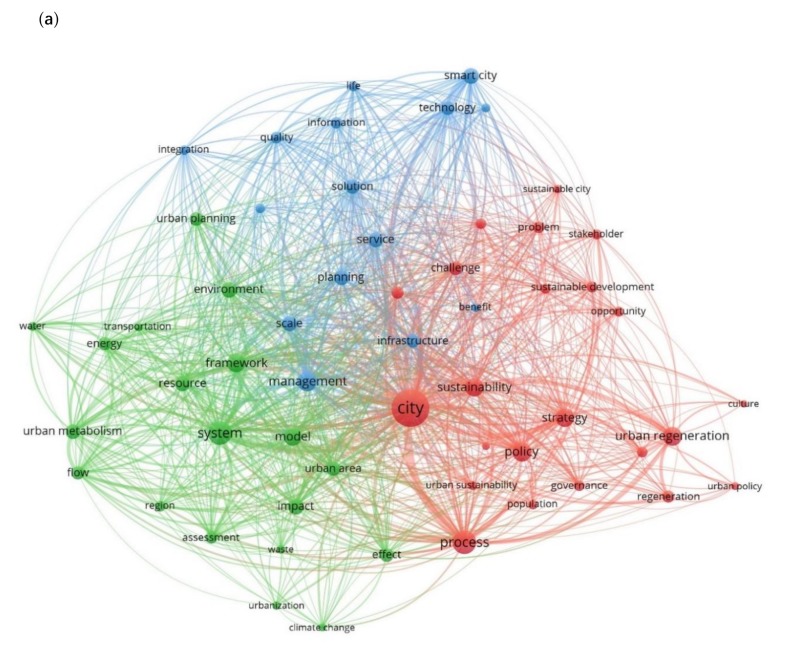

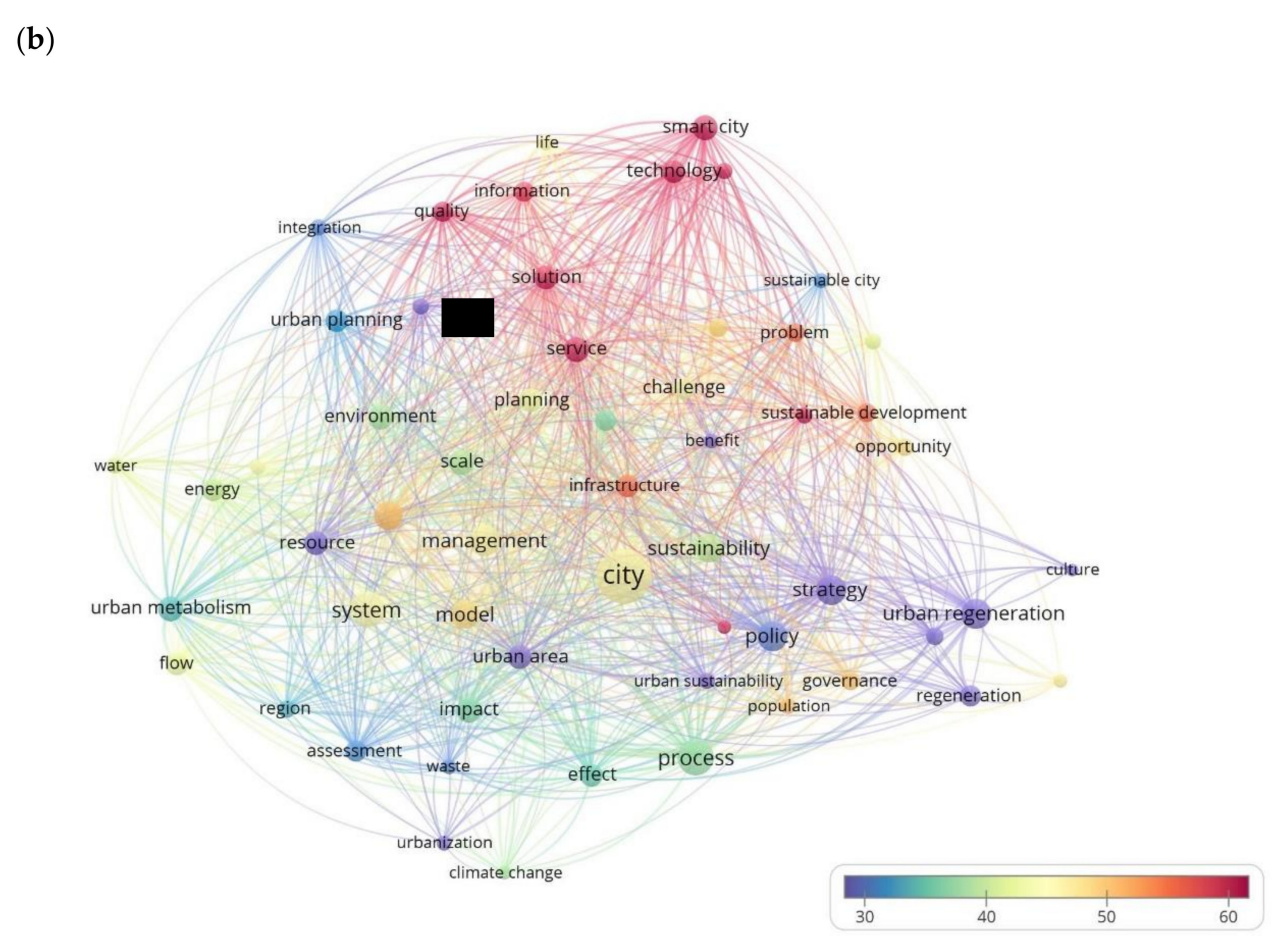
(**a**). Map of co-occurrence analysis based on keywords. (**b**). Map of co-occurrence analysis based on keywords indicating the average of citation.

The first map produced presents a bibliographic network of 57 nodes/keywords connected with co-occurrence links and grouped into three clusters (Figure 4a, Table 2). This first part of this analysis shows the keywords that appear together and their frequency in the data set. Their proximity to another reveals the relatedness of each pair of terms. The color of each node indicates the cluster in which it belongs. These keywords clusters can be interpreted as the research trends of the topic under review.

Looking at the links between the keywords we identify that the top five pairs of keywords with the greatest co-occurrence are *city* and *process* with link strength equaling to 44, *city* and *system* with link strength of 43, *city* and *strategy* with link strength of 30, *city* and *smart city* with link strength of 28, *city* and *management* with strength of 26. Our general understanding from these links is that the most connected/related part of the literature treats cities as systems, and under system analysis the literature studies the associated processes seeking for strategies to ensure a smart and more efficient urban management.

In Table 2, we can see all the keywords per cluster and how many times they appear in the data set (number of occurrences). In the first cluster (red color), which contains 23 keywords, the top five keywords with the greatest number of occurrences are *city* which appears 143 times; *urban regeneration* 52 times; *policy* 51; *strategy* 50 times; and *sustainability* 49 times. The second cluster (green color), which contains 19 keywords, denotes a more engineering approach as the five terms with the higher occurrence, are *system* 71 times; *framework* 48 times; *model* 47 times; *impact* 39 times; and *environment* 38 times. The third cluster (blue color) of 15 keywords presents mixed terms from different scientific fields since the top five terms are *management* 53 times; *smart city* 38 times; *scale* and *service* 36 times each; and *planning* 35 times.

Figure 4b and Table 3 present the results of the second part of this analysis. We can see the co-occurrence network of keywords weighted by the number of occurrences of each keyword and colored considering the average number of citations of the documents that these keywords have. Looking at Table 3, we see the exact number of average citations that corresponds indirectly to each keyword per cluster. In the first cluster, the five first keywords with the greatest number of average citations are *knowledge* (100.11), *sustainable development* (54.70), *problem* (54.46), *population* (50.44), and *governance* (50.05). In the second cluster, the keywords *framework* (50.58), *model* (48.45), *system* (45.38), *transportation* (44.88), and *water* (41.71) are the five keywords that occurred in documents with greatest average citations. In the third cluster, these keywords are *smart city* (91.68), *citizen* (89.65), *technology* (88.41), *service* (74.25) and *quality* (64.76).

Based on this analysis and looking at the number of occurrence and average citations, we understand that the literature is divided into three main research trends (clusters) of urban growth. The first research trend tries to understand the urban processes and to apply this knowledge in order to tackle related urban challenges and problems. This part of the literature is seeking policies and strategies that support sustainable urban development, offering opportunities for urban regeneration involving different stakeholders. The second research trend studies urban systems, on a regional scale using an urban metabolism framework to tackle the negative environmental impacts of these urban systems. In this way, we model the consumption of resources, the flows of energy and material within urban systems (i.e., water, transportation), and the resulting outcomes to other systems in the form of pollution, waste or export product. The third research trend refers, to the integration of the concept “smart city” in urban planning and management at different scales of analysis. Smart city concept benefits the citizens by increasing the overall quality of life offering solutions, using technological infrastructures to have access to services and information.

**Figure 5:**
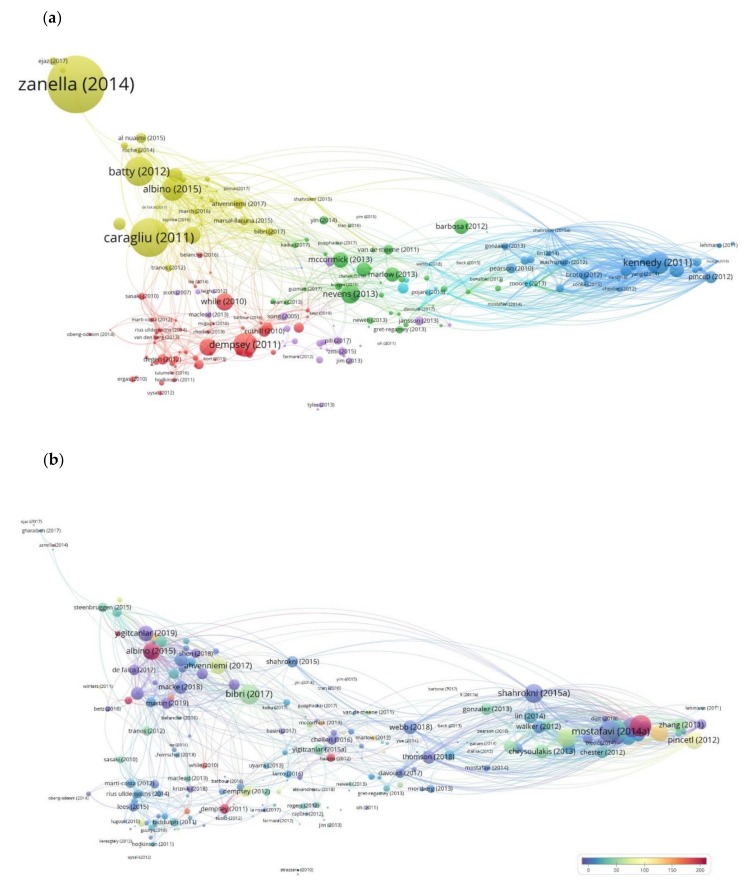
(**a**). Map of bibliographic coupling analysis based on documents with the weights of the links on the number of citations. (**b**). Map of bibliographic coupling analysis based on documents with the weights of the links on the total links strength and indicating the number of citations.

Figure 5a,b present the bibliographic coupling network of the documents, our second analysis. As shown in Figure 5a, the network of 228 nodes represents the connected documents of our data set under this analysis, and it is grouped into six clusters. The size of the labels of the documents represented by circles varies depending on the number of citations referring to the documents (weight) (Appendix Table A1, and Table A3). The top five cited documents in the first cluster of 60 items (red color) are Dempsey et al. (2011) 275 times cited, While et al. (2010) 172 times cited, González et al. (2013) 166 times cited, Cuthill (2010) 91 times cited, and Degen & Garcia (2012) 79 times cited. In the second cluster of 47 items (green color), the top five documents with more citations are Nevens et al. (2013) with 191 citations, McCormick et al. (2013) with 144 citations, Barbosa et al. (2012) with 135 citations, Marlow et al. (2013) with 126 citations, and Zhao (2010) with 121 citations. For the cluster three (blue color) of 40 items Kennedy et al. (2011) 258 times cited, Chen & Chen (2019) 127 times cited, Pincetl et al. (2012) 98 times cited, Barles 2010) 86 times cited and Pearson et al. (2010) 74 times cited are the top highly cited documents. The most cited documents of the fourth cluster of 40 items (yellow color) are Zanella et al. (2014) with 1065 citations, Caragliu et al. (2011) with 576 citations, Batty et al. (2012) with 372 citations, Albino et al. (2015) with 260 citations and Lombardi et al. (2012) with 158 citations. For the fifth cluster (purple color) of 31 items the top five cited documents are Haapio (2012) 88 times cited, Yigitcanlar & Lee (2014) and Jansson (2013) with 60 times cited each, Zitti et al. (2015) with 58 times cited and Pili et al. (2017) with 51 times cited. For the sixth cluster of 10 items (light blue color), Haghshenas & Vaziri (2012) with 80 citations, Moore et al. (2013) with 56 citations, Pojani & Stead (2015) with 40 citations, Liu (2012) with 30 citations, and Newton & Glackin (2014) with 19 citations are the top five cited documents. The results of this analysis show the scholarly sources with a higher impact in the general field of urban and environmental planning. These sources constitute publications with a greater number of citations.

In the second part of this analysis shown in Figure 5b, we see the network of the key scholarly sources based on their relevance to the topic under analysis. The size variation of the labels of the nodes is determined by their total link strength, which here is the number of common documents that they cite with the documents on the other edge of the links. On the other hand, the differentiation in color is based on the number of citations of each document. Looking at Table 4, we see the top five more important scholarly sources per cluster considering their total link strength, as well as the sub research trends per cluster in titles.

The first cluster introduces the social dimension to sustainable urban development; in this way, urban sustainability refers not only to the environmental concerns but also includes social and economic aspects to the concept. Social equity, environmental equity, and the sustainability of the community itself are the main dimensions of social sustainability. Social equity relates to the access to services and facilities, environmental equity relates to the access to green and open spaces, and the sustainability of community includes perceptions of safety, social interaction, and community stability. Overall, social sustainability is seeking social cohesion, capital inclusion, and high quality of the living environment. A question raised here is if high-density neighborhoods support less social sustainability than the low-density ones. Findings reveal that denser areas provide access to services and facilities at the neighborhood level, but the use of them depends on their quality. Regarding the aspect of environmental equity, urban denser areas appear to offer less public green open spaces than the low-density urban areas. Furthermore, residents chose or not to use the local green areas according to the feeling of safety and the level of maintenance of the site. It appears that in higher-density neighborhoods, the local parks and green spaces are less attractive and unsafe than in the lower-density areas. From a community stability and social interaction point of view, high-density areas appear less stable with residents expressing the feeling of lower satisfaction with their neighborhood and tendency to move somewhere else. Moreover, high-density areas seem to have weaker social interaction and social networks than the lower-density areas. A solid way to combat social exclusion is though urban regeneration supported by arts and culture. Artists using ethnographic methods can challenge the links between regeneration and gentrification. This new form of economy based on culture, creativity, and knowledge makes cities unique avoiding standardization and by giving them authenticity, it turns them into globally competitive cities.

There is a lack of greater knowledge about patterns of urbanization and the types of urban areas over time and space. It is, however, known that the contemporary urbanization in both low/middle-income and high-income countries differs from the historical urbanization and coevolution of urban areas. The dynamics of the built environment and socio-institutional and natural systems show constraints and alternative opportunities not viewed in the earlier urbanization patterns. Nowadays, urban areas originate negative environmental consequences associated with carbon flows, energy demands, waste, air pollution, and noise pollution, loss of biodiversity. At the same time, since urban areas constitute the basic units for policies, they have environmentally beneficial consequences in three scales; global, local, and individual environmental behavior. Thus, rapid urbanization can accelerate a transition to sustainability due to agglomeration, increased innovation, and increased wealth, requiring suitable governance structures. The lack of a standardized definition for an urban area is challenging the scholars, shifting the focus from politico-administrative boundaries to physical or geomorphological boundaries, and leading to the need to rethink the sustainable urban development concept and practice towards an integrated planning and development process. The process can be achieved by integrating the systematic rational urban planning approach with ecosystem sustainability to increase the livability of urban areas and maintain the existence of urban ecosystems. One attempt to include ecological principles to urban planning is the regenerative urban metabolism systematic approach to conduct a quantitative analysis of human activities and land use. Also, the integration of people-centered and top-down approaches is required to enhance urban metabolism participation management when addressing urban sustainability transitions. People-centered approaches could be related to both urban metabolism management facets, via the participation within decentralized or inverse infrastructures management or contributing to leveraging behavioral changes in resources consumption. The urban metabolic facets are the *built facet*, which includes the physical infrastructures and the intangible *facet* meaning the services, resources, and flows of consumption.

The third cluster describes the evolution of the urban metabolism concept seeking a sustainable urbanized planet. In 2007 C. Kennedy, following Wolman´s work, updates urban metabolism as “the sum total of the technical and socio-economic processes that occur in cities, resulting in growth, production of energy, and elimination of waste.” The concept has been used from two different schools; the first one focuses on the energy equivalents (influenced by the work of Odum), and the second one tries to describe urban metabolism using a broader approach uttering the flows of water, materials, and nutrients as mass fluxes. Successive generations of urban metabolism are identified based on analytical methods. The earliest generation uses mainly Material Flow Analysis (MFA) measuring the material fluxes into the urban systems, the stocks and flows within the systems, and the resulting outcomes to other systems in the form of pollution, waste, or export products. The second urban metabolism generation uses the Energy method and the Ecological Footprint (EF), moving the focus beyond mass. The most recent urban metabolism generation couples urban metabolism with Life Cycle Assessment (LCA) with Integrated Urban Metabolism Analysis Tools (IUMATs). Integrated urban metabolism tries to simulate the inter-dependencies between the variables and subsystems of an urban region to compute the urban environmental performance. Since the first mention of urban metabolism in urban ecology history, the concept reveals six themes in context of interdisciplinary synergies: 1) the city as an ecosystem; 2) the material, and energy flows in the city; 3) the economic drivers of rural-urban relationships; 4) the material basis of the economy; 5) the reproduction of urban inequality and the re-signifying of the city via new socio-ecological relationships, and finally 6) the hybrid nature-socio-eco-politic urban metabolism of the last generation considers the dynamics of choice, time, and scale for the plan and design of sustainable urban areas.

Cluster four examines the concept of smart and sustainable cities by looking at their similarities and differences, and the overall contribution of smart city concept to the goals of sustainable urban development to answer the main question if cities can be smart without being sustainable. The concept of “smart city” is a fuzzy concept that has been used since the 1990s with many definitions, all of them far from just the application of technologies to cities. There are two successive main parts of smart city literature. The first one focuses principally on the technical and environmental aspects of a city applying modern technologies in daily urban life for a better quality of life, decreasing the environmental impacts. The second part adds to that the variable of human capital in developing smart cities, “as a holistic understanding that smart cities bring together technology, government, and society.” In other words, there is the (ICT)/technology-oriented approach and the people-oriented approach. The goal of sustainable urban development is to “achieve a balance between the development of the urban areas and protection of the environment with an eye to equity in income, employment, shelter, basic services, social infrastructure and transportation in the urban areas,” to create healthy, livable, and prosperous human environments minimizing the demand of resources and the environmental impacts. Smart cities with a holistic understanding of the investment in human, social, and environmental capitals generate urban sustainability. Thus, cities cannot be truly smart if they are not sustainable, so the term “smart sustainable city” instead of smart city is suggested.

Cluster five highlights the need to include ecological processes to the analysis and assessment of urban systems under a sustainable urban development concept. Urban policy and planning must emphasize ecosystem functions and services to sustain biodiversity in urban landscapes. Tools and models such as urban metabolic models, land-use modeling, transportation models, and urban growth models can integrate both urban systems and ecosystems. Merging the concepts of “ecology in cities” and “ecology of cities” in combination with the ecosystem services framework, we as urbanites acknowledge the city as an ecosystem that depends on its surrounding landscape. In this way, human development is reconnecting to the biosphere and ecosystem services. The final step to establish sustainable future urbanization is to operationalize this knowledge of the socio-ecological interconnections, by translating the work of biodiversity into ecosystem services and the quantification of resilience. One good example of this reconnection is this of TEBEE that gives economic value to ecosystem services. Sustainability is an elastic concept that can be weak when it relies on technological fixes with little change of individual behaviors and lifestyle towards sustainability, and strong when changes are applied on the three dimensions of sustainability; economy, society and environment using advanced technologies. This new urbanism movement, focusing on the regeneration of the urban environment, converges with smart growth concept under the principle of sustainable development and branding cities as low-carbon, carbon-neutral, smart sustainable, smart eco-city, and ubiquitous eco-city (u-eco-city). Moreover, this new urbanism movement is now re-directing research to answering the following key-question: who is economically benefitting the most, if there is true e-democracy, true quality of life, if a city is treated as a whole, including citizens’ voice in city planning and management.

Cluster six explores the regenerative design of urban areas using the urban metabolism framework. Urban metabolism framework quantifies the consumption of energy and materials and helps to compare the ecological footprint of this consumption. Furthermore, using the LCA method, it captures the hidden fluxes of energy and materials associated with the manufacturing of various products. Cities as complex social-ecological-technological systems require a holistic multi-scale approach of co-design and co-production of knowledge to support urban policy and development. This knowledge framework helps to understand how urban systems behave and evolve, how different urban fabrics and urban profiles determine the urban resource flows having different urban metabolisms. The next step is to apply this knowledge and to implement the methodology, by redesigning a city, in a regenerative way, to reduce its ecological footprint. A regenerative city environmentally enhances and restores the relationship between the cities and the natural systems they depend on. Urban regeneration based on decentralized energy systems allows mixing different renewable systems for energy generation and supply. Moreover, it offers new lifestyle choices and economic opportunities, which lead to long-term community involvement in this transformation process. Thus, the regenerative urban metabolism framework offers solutions to megatrends such as climate change through energy use reduction, resource scarcity through efficient material use, biodiversity loss, and urban encroachment on rural areas through compact city footprints

**Figure 6:**
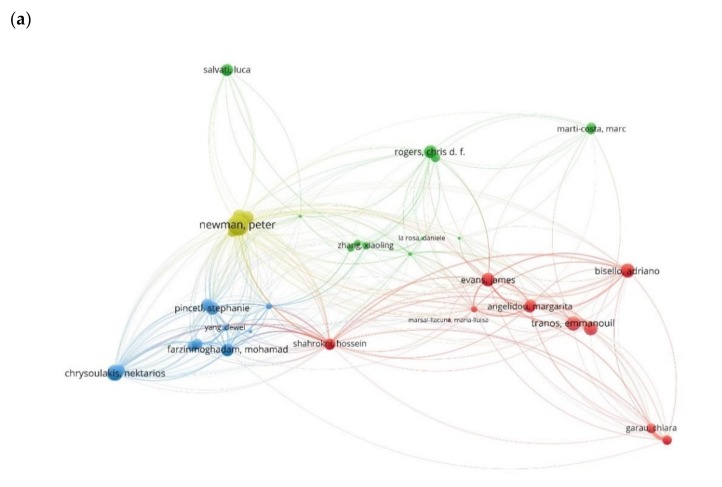

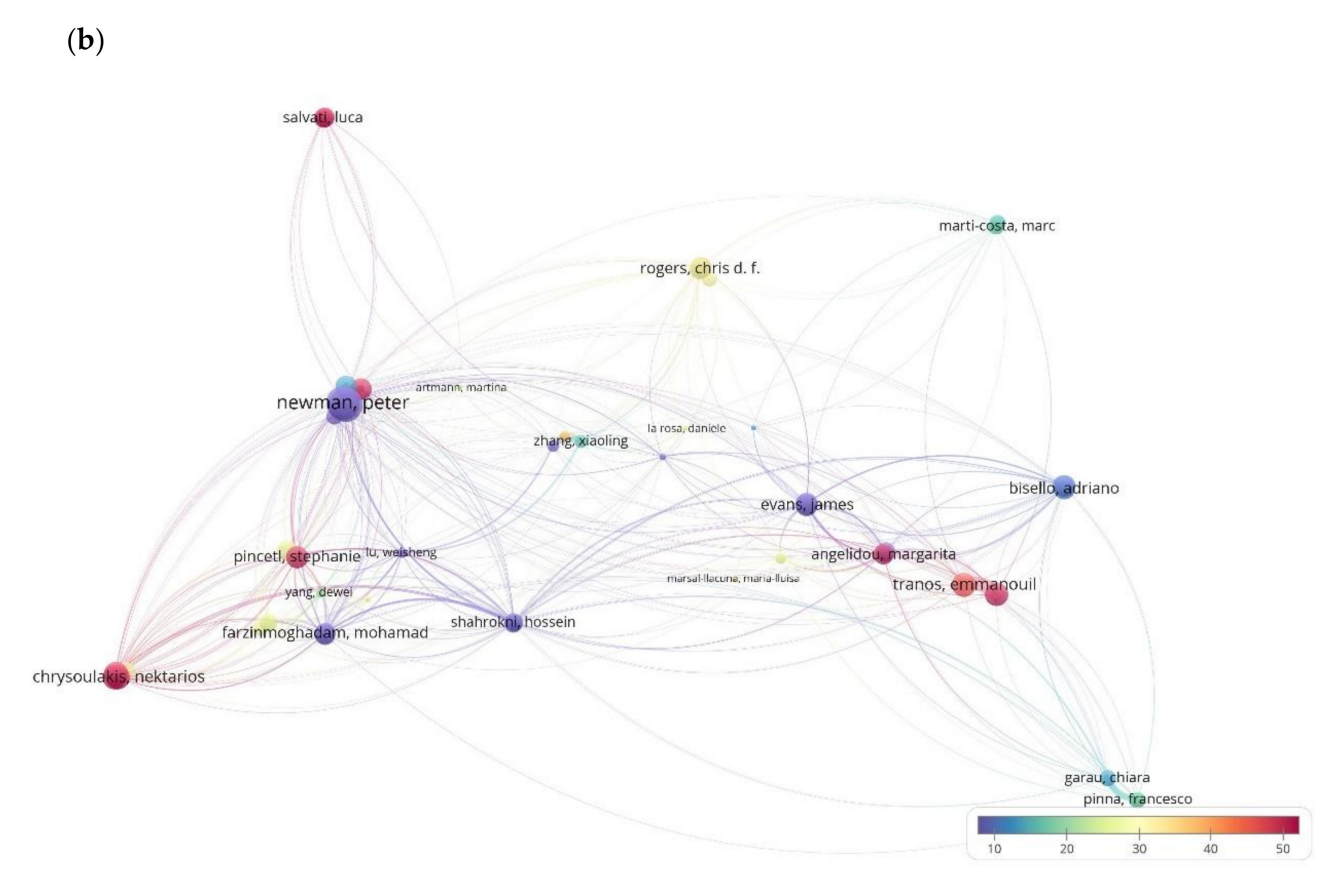
**(a).** Map of bibliographic coupling analysis based on authors (weights on the total link strength).(**b**). Map of bibliographic coupling analysis based on authors with the weight on the number of documents and indicating the number of average citations.

From the third analysis, we created a map of 49 bibliographic coupled authors grouped in four clusters (Figure 6a). In this map, we can see which authors have stronger bibliographic coupling links between each other. By looking at the size variation of the links (thinker the line of the link stronger connection between authors) and at the size variation of the nodes we can see which authors have the total stronger bibliographic coupling links in the literature network. In this way, we detect which authors share similar ideas and or influence each other. The top five stronger connected authors per cluster are mentioned in Table 5. Looking at Appendix Table A1, the articles found in dataset understudy and combining this with the knowledge obtained from previous analyses, we conclude that authors in the first cluster share ideas based on the sub-research trends present in cluster 1 and 4 of our second analysis (Table 4), authors in the second cluster study sub-research trends found in cluster 1 and 5 (Table 4), the work of authors in the third cluster is based on cluster 3 of the second analysis (Table 4) and authors in the fourth cluster study the main topics found on cluster 2 and 6 in the second analysis (Table 4).

In Figure 6b, the color variation of the nodes of the network shows the average citation of each author under bibliographic coupling analysis. The average citation and the total link strength for each author are found in Table A2. We see the top five authors with the greatest number of average citations for cluster 1 are *Nikjamp Peter* (309.0), *Angelidou Margarita* (62.50), *Tranos Emmanuil* (45.00), *Maria Luis* (34.50), and *Yigitcanlar Tan* (27.00). In the second cluster the authors *Carlucci Margherita* (54.50), *Salvati Luca* (54.50), *Lombardi D. Rachel* (37.00), and *Wang Rusong* (36.50) are the five top authors with the greatest number of average citations, in the third cluster, these authors are *Pincetl Stephanie* (51.67), *Chrusoulakis Nektarios* (50.00), *Lopes Myriam* (50.00), *Rosado Leonardo* (33.00), and *Spano Donatella* (31.50), and in the four cluster the authors *Moglia Mangus* (68.50), *Newton Peter* (15.00), *Davoudi Simin* (7.00), *Newman Peter* (15.00), and *Thomson Giles* (6.33).

**Figure 7:**
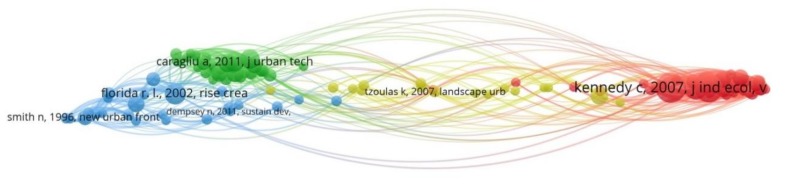
Map of co-citation analysis based on cited references.

In our last analysis, we constructed the co-citation map of the 117 connected cited references of our data set, grouped in four clusters (Figure 7). On this map, we can identify the connections-links of the cited references that have been cited jointly by another document, which allow us to infer about the relativeness importance of the document, i.e., the more cited, the higher the importance of the document. In this analysis the strength of the links (weights) represents the number of citations made to the cited reference of each node. In Table 6, we can see the top five cited references per cluster, their links with other cited references of our data set, and their number of citations. Knowing the most influential cited references of the documents under review per infometric cluster, we can detect which authors have been influenced by whom and how specific concepts have been formed and evolved over the years. We can also go back to study the original ideas and draw a concept evolution timeline.

## 4. Discussion

### 4.1. Discussion and Limitations of Methodology

The novelty of the proposed methodology of literature network analysis in comparison with the traditional way of conducting a literature review is the fact that it is using bibliographic metrics. Therefore, we can distinguish that this analysis has a threefold objective. Firstly, it is pedagogical. Secondly, it eliminates the uncertainty of a randomly casual literature review and reduces complexity by mitigating all the noise of the big volume of data accessible through the internet. Thirdly, it is methodological, thus it provides a literature review with coherence and a scientific protocol to acquire oriented-knowledge. The undertaken literature network analysis is detailed and descriptive, discussing the planning stages and explaining the operational steps of the literature review. Finally, the obtained results are illustrated through tables and distance maps, providing insights for scholars who are beginning their research on the topic avoiding an initial random search.

It is essential to clarify that these bibliographic networks show the relatedness of the items under study based on how strong the links that they share are. In bibliographic coupling networks, links exist between items that cite the same document, in co-citation between items that they have been cited by the same document, in co-occurrence networks regarding the number of documents in which they occur together. Thus, from a technical point of view, this method demonstrates effectiveness. Nevertheless, limitations are stemming from the interpretation of these networks in the cluster analysis stage. For instance, in case of co-occurrence networks, keywords can occur together in more that one paper having different meanings and thus generating misleading bibliographic networks. In bibliographic coupling networks, two items can cite the same document but expressing disagreement about the topic under study. In order to diminish these issues in the network analysis, we carefully selected the initial literature dataset based on multiple group discussions by experts. Other limitations arise from the methods applied to conduct network analysis. As mentioned in a previous section, there are two counting methods, the full and fractional counting methods. A researcher has to be aware of the limitations arising from the different methods applied in various networks. For instance, using fractional counting method, highly cited articles that have a smaller influence on the construction of bibliographic coupling networks and articles with many references like review articles, have a less important role in the construction of co-citation networks. Articles with many authors have the same weight with articles with less authors in the construction of co-authorship networks [25,26]. We used fractional counting method when we wanted to give equal importance to the items under network analysis. Despite the identified limitations, it is our understanding that literature network analysis provides a faster rate of discovery, more accurate and more in-depth insights than other literature review methods we have known, and experimented with until now.

### 4.2. Discussion of Results

**Figure 8:**
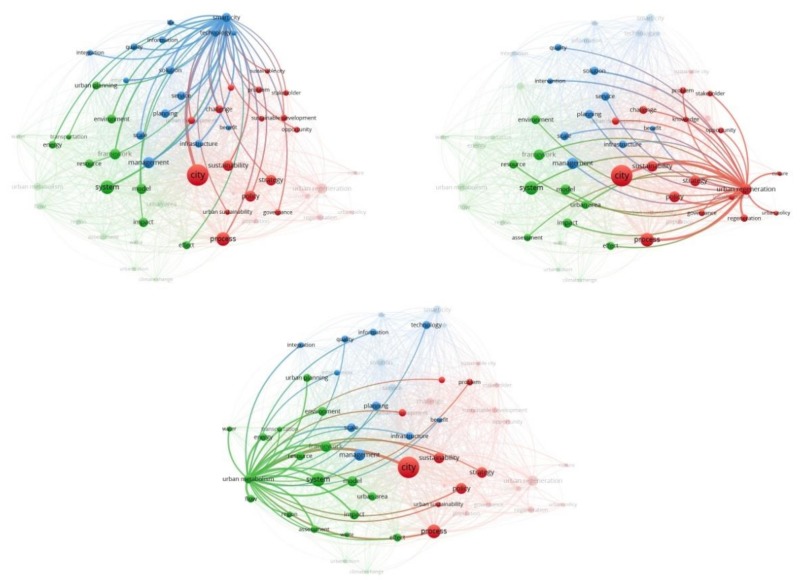
Links between main keywords under co-occurrence analysis.

A general observation from all the different types of bibliometric analysis we conducted here is that the number of citations cannot be taken as the main driver to show the importance of an item without taking into consideration the total link strength of the item; meaning the degree of connection with the rest of the items in the dataset under analysis. For instance, comparing the two maps (Figure 5a,b), we see that documents with an extremely high number of citations are not the best connected to the rest of the documents of the data set, and therefore they are not the most related to the topic of analysis. Looking at the links of the items of all our bibliometric analysis, we observe that the links between the concepts of smart and regenerative metabolic urban growth do not appear to be strong, or appear to be absent. As an example, shown in Figure 8, we have made a selection of the keywords of our interest *smart city*, *urban metabolism*, and *urban regeneration*, and can clearly see that they are not connected.

**Figure 9:**
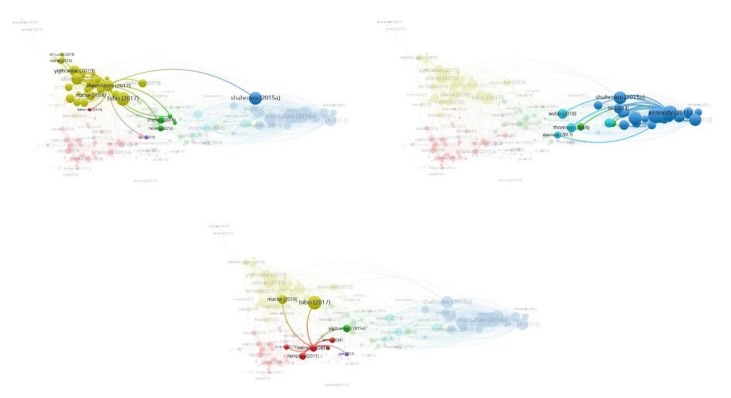
Links between main concepts under bibliographic coupling analysis based on documents.

We obtain the same image when looking closely at the links in our second analysis of the bibliographic coupling of documents (Figure 5a). In Figure 9, we have selected one representative document of each concept smart city; Ahvenniemi et al. (2017), urban metabolism; Dempsey et al. (2012), urban regeneration; Kennedy et al. (2011) to make this statement better understood.

## 5. Conclusions

**Figure 10:**
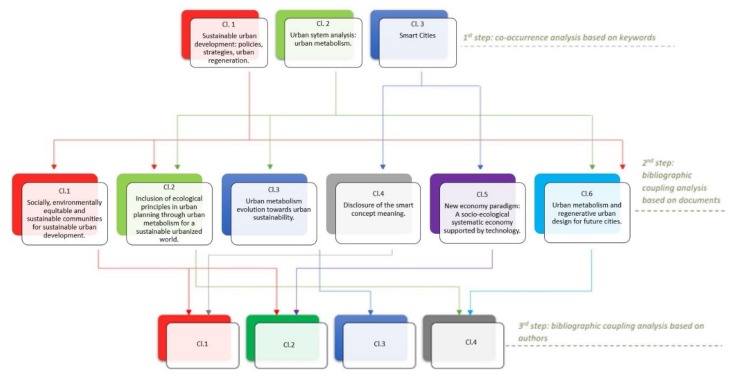
Map of cascade relations among the bibliometric clusters.

In this study, we conducted a literature network analysis to review the concept of urban growth under metabolic framework focusing on smart and regenerative urban design within the last ten years. Using VOSviewer we constructed network maps that helped us detect relatedness of concepts, documents, main referenced works, and top influencers authors along with tip-top cutting-edge research upon the topic. Initially, we indicated three main research trends related to urban growth (see results of analysis 1) and going one step further, we were able to identify six key sub-research trends (see results of analysis 2) and their relatedness. We detected the most influencer authors within our dataset per sub-concept (see results of analysis 3) (Figure 10), and finally, we tracked the origins of these key sub-research trends related to the urban growth concept under analysis (see results analysis 4). The overall findings showed that urban growth research is simultaneously multidisciplinary and interdisciplinary integrating social, ecological, politic, economic sciences, culture and arts, environmental, and computer sciences. We identified a lack of connectedness between smart and regenerative concepts for urban growth. Therefore, this provides scientific evidence that in order to adopt a holistic approach allowing future cities to tackle challenges related to unbalanced urbanization-economy-environment dynamics, and to provide a better life quality and wellbeing, we need to turn the research focus on building this link between the fields of smart and regenerative urban studies. We have already started to conceptualize the framework of smart and regenerative urban growth in post anthropocentric urbanism.

## Figures and Tables

**Table 1:** Number of occurrences of keywords ( K_1-3_) in documents (D_1-5_).

Documents					
Keywords/Terms	D1	D2	D3	D4	D5
**K1**	1	2	3	4	5
**K2**	1	2	3	1	2
**K3**		1	1	2	

**Table 2:** Number of co-occurrences of the selected keywords per cluster.

CLUSTER 1 (23 Items)		CLUSTER 2 (19 Items)		CLUSTER 3 (15 Items)	
Keywords	No. Occurrences	Keywords	No. Occurrences	Keywords	No. Occurrences
Challenge	32	Assessment	27	Benefit	16
City	143	Climate change	14	Citizen	17
Culture	12	Effect	32	Information	25
Governance	22	Energy	30	Infrastructure	30
Knowledge	19	Environment	38	Integration	18
Opportunity	20	Flow	30	Intervention	18
Policy	50	Framework	48	Life	19
Population	18	Impact	39	Management	53
Problem	24	Model	47	Planning	35
Process	72	Region	21	Quality	25
Regeneration	27	Resource	35	Scale	36
Stakeholder	19	System	71	Service	36
Strategy	50	Transportation	16	Smart city	38
Sustainability	49	Urban area	34	Solution	33
Sustainable city	15	Urban metabolism	36	Technology	32
Sustainable development	23	Urban planning	31		
Sustainable urban development	22	Urbanization	15		
Transformation	21	Waste	15		
Urban development	27	Water	17		
Urban environment	14				
Urban policy	14				
Urban regeneration	52				
Urban sustainability	17				

**Table 3:** Average citations of the selected keywords per clusters in co-occurrence analysis.

CLUSTER 1 (23 Items)		CLUSTER 2 (19 Items)		CLUSTER 3 (15 Items)	
Keywords	Avg. Citations	Keywords	Avg. Citations	Keywords	Avg. Citations
Challenge	44.22	Assessment	32.37	Benefit	26.88
City	46.08	Climate change	39.07	Citizen	89.65
Culture	26.17	Effect	35.66	Information	57.72
Governance	50.05	Energy	40.57	Infrastructure	54.53
Knowledge	100.11	Environment	39.16	Integration	31.89
Opportunity	48.35	Flow	43.23	Intervention	15.78
Policy	31.31	Framework	50.58	Life	45.42
Population	50.44	Impact	37.10	Management	46.09
Problem	54.46	Model	48.45	Planning	43.77
Process	38.00	Region	34.24	Quality	64.76
Regeneration	21.15	Resource	23.36	Scale	39.42
Stakeholder	42.84	System	45.38	Service	74.25
Strategy	26.54	Transportation	44.88	Smart city	91.68
Sustainability	40.37	Urban area	25.09	Solution	59.06
Sustainable city	32.40	Urban metabolism	34.92	Technology	88.41
Sustainable development	54.70	Urban planning	33.03		
Sustainable urban development	48.77	Urbanization	30.13		
Transformation	25.81	Waste	31.93		
Urban development	37.44	Water	41.71		
Urban environment	72.71				
Urban policy	46.79				
Urban regeneration	15.17				
Urban sustainability	19.76				

**Table 4:** Top five publications per infometric cluster with stronger links based on bibliographic coupling analysis. References can be found in Appendix Table A3.

	No. Citations	No. Links	Total Link Strength	Important Publications	Main Topics of the Clusters
**CLUSTER 1** **(60 Items)**	76	38	87	Dempsey et al. (2012)	Socially, environmentally equitable, and sustainable communities for sustainable urban development.
	12	35	83	Lees and Melhuish (2015)	
	275	40	82	Dempsey et al. (2011)	
	23	32	72	Martí-Costa and Miquel (2011)	
	22	35	70	Rius Ulldemolins (2014)	
**CLUSTER 2** **(47 Items)**	3	56	93	Yigitcanlar and Teriman (2015)	Inclusion of ecological principles in urban planning through urban metabolism for a sustainable urbanized world.
	26	44	87	Romero-Lankao et al. (2014)	
	14	41	81	Lu et al. (2016)	
	2	62	80	Chelleri et al. (2016)	
	191	36	62	Nevens et al. (2013)	
**CLUSTER 3** **(40 Items)**	7	53	282	Mostafavi et al. (2014a)	Urban metabolism evolution towards urban sustainability.
	258	46	266	Kennedy at el. (2011)	
	42	52	252	Goldstein et al. (2013)	
	1	50	232	Zhang et al. (2018)	
	70	55	222	Broto et al. (2012)	
**CLUSTER 4** **(40 Items)**	58	78	242	Bibri and Krogstie (2017)	Disclosure of the smart concept meaning.
	260	52	188	Albino et al. (2015)	
	77	55	177	Ahvenniemi et al. (2017)	
	0	46	162	Yigitcanlar et al. (2019)	
	8	56	127	Macke et al. (2018)	
**CLUSTER 5** **(31 Items)**	17	48	62	Mortberg et al. (2013)	New economy paradigm: A socio-ecological systematic economy supported by technology.
	40	44	52	Lombardi et al. (2011)	
	37	32	49	MacLeod (2013)	
	60	31	39	Jansson (2013)	
	60	19	36	Yigicanlar and Lee (2014)	
**CLUSTER 6** **(9 Items)**	11	73	138	Webb et al. (2018)	Urban metabolism and regenerative urban design for future cities.
	4	64	127	Thomson and Newman (2018)	
	56	51	124	Moore et al. (2013)	
	10	54	82	Davoudi and Sturzaker (2017)	
	6	40	45	Van Timmeren et al. (2012)	

**Table 5:** Most connected authors per infometric cluster.

	Link Strength	Most Connected Authors
**CLUSTER 1** **(17 Items)**	151.35	Tranos Emmanouil, Nijkamp Peter
	118.58	Evans James, Karvonen Andrew
	99.80	De Falco Stefano, Angelidou Margarita
	66.86	Mosannenzadeh Farnaz, Bisello Adriano, Vettorato Daniele
	40.91	Lazarevic David, Brandt Nils, Shahrokni Hossein
**CLUSTER 2** **(14 Items)**	118.20	Carlucci Margherita, Salvatti Luca
	90.77	Lombardi Rachel, Rogers Chris D. F.
	88.53	Pares Marc, Marti-Costa Marc
	40.23	Porter Libby, Rogers Chris D. F.
	39.90	Porter Libby, Lombardi Rachel
**CLUSTER 3** **(13 Items)**	107.80	Chester Mikhail, Pincetl Stephanie
	99.64	Farzinmoghadam Mohamad, Mostafavi Nariman
	77.82	Zhang Yan, Liu Gengyuan
	61.44	Lopes Myriam, Chrysoulakis Nektarios, Gonzalez Ainhoa
	32.60	Spano Donatella, Lopes Myriam, Gonzalez Ainhoa, Chrysoulakis Nektarios
**CLUSTER 4** **(40 Items)**	142.43	Thomson Giles, Newman Peter
	44.36	Newton Peter, Thomson Giles
	44.36	Newton Peter, Newman Peter
	43.61	Moglia Mangus, Thomson Giles
	43.61	Moglia Mangus, Newman Peter

**Table 6:** Important cited references under co-citation links per infometric cluster.

	No. Cit.	No. Links	Important Cited References
**CLUSTER 1** **(43 Items)**	29	63	Kennedy, C.A., Cuddihy, J., Engel Yan, J., 2007. The changing metabolism of cities. Journal of Industrial Ecology 2007 (11), 43-59
	26	53	Wolman, A., 1965. The metabolism of cities. Scientific American 213 (3), 179-190
	20	55	Newman, P.W.G., Birrell, R., Holmes, D., Mathers, C., Newton, P., Oakley, G., O’Connor, A., Walker, B., Spessa, A., Tait, D., 1996. Human settlements. In: Australian State of the Environment Report. Department of Environment, Sport and Territories, Canberra, Australia.
	18	51	Kennedy, C., P. Pincetl, and P. Bunje. 2011. The study of urban metabolism and its applications to urban planning and design. Environmental Pollution 159(8–9): 1965–1973.
	12	46	Niza, S., L. Rosado, and P. Ferrao. 2009. Urban metabolism: Methodological advances in urban material flow accounting based on the Lisbon case. Journal of Industrial Ecology 13(3): 384–405.
**CLUSTER 2** **(34 Items)**	15	58	Hollands, R.G., 2008. “Will the Real Smart City Please Stand Up?” City: Analysis of Urban Trends, Culture, Theory, Policy, Action 12: 3, 303–320.
	15	57	Caragliu, A., Del Bo, C., & Nijkamp, P. (2009). Smart cities in europe, serie researchmemoranda 0048. VU University Amsterdam, Faculty of Economics, BusinessAdministration and Econometrics.
	14	51	Giffinger, R., Fertner, Ch, Kramar, H., Kalasek, R., Pichler-Milanovic, N., et al. (2007). Smart cities-ranking of European medium-sized cities. Centre of RegionalScience (SRF), Vienna University of Technology.
	13	49	Vanolo, A. (2014). Smartmentality: The smart city as a disciplinary strategy. Urban Studies, 51, 883–898.
	11	46	Neirotti, P., De Marco, A., Cagliano, A. C., Mangano, G., & Scorrano, F. (2014). Current trends in smart city initiatives: Some stylised facts. Cities, 38, 25–36.
**CLUSTER 3** **(24 Items)**	17	33	Florida, R. (2002) The rise of the creative class. Basic Books, New York.
	12	26	Harvey D (1989) From managerialism to entrepreneurialism: The transformation in urban governance in late capitalism. Geografiska Annaler: Series B, Human Geography 71(1): 3–17.
	10	13	Smith, Neil (1996). The new urban frontier. Gentrification and the revanchist city. London: Routledge.
	9	35	Florida, R (2005). Cities and the Creative Class. Routledge, New York.
	7	17	Peck, J (2005) Struggling with the creative class. International Journal of Regional Research 29(4), 740–770.
**CLUSTER 4** **(16 Items)**	13	35	Grimm, N. B., Faeth, S. H., Golubiewski, N. E., Redman, C. L., Wu, J., Bai, X., et al. (2008). Global change and the ecology of cities. Science, 756–760.
	7	16	Tzoulas, K., Korpela, K., Venn, S., Yli-Pelkonen, V., Kaźmierczak, A., Niemela, J., & James, P. (2007). Promoting ecosystem and human health in urban areas using Green Infrastructure: A literature review. Landscape and Urban Planning, 81(3), 167–178.
	6	37	Campbell, S. (1996). Green cities, growing cities, just cities? Urban planning and the contradiction of sustainable development. Journal of the American Planning Association, 62, 296–312.

## Appendix A

**Table A1:** Number of citations of documents per cluster based on the bibliographic coupling analysis.

CLUSTER 1 (60 Items)		CLUSTER 2 (47 Items)		CLUSTER 3 (40 Items)		CLUSTER 4 (40 Items)		CLUSTER 5 (31 Items)		CLUSTER 6 (10 Items)	
Documents Citations		Documents Citations		Documents Citations		Documents Citations		Documents Citations		Documents Citations	
Arbaci (2012)	31	Alexandrescu et al. (2018)	6	Barles (2010)	86	Ahvenniemi et al. (2017)	77	Caputo et al. (2012)	13	Davoudia and Sturzakerb (2017)	10
Baba (2017)	2	Artmann (2014)	20	Basiri et al. (2017)	3	Al Nuaimi et al. (2015)	78	Dell‘ollo et al. (2014)	10	Haghshenas and Vaziri (2012)	80
Bailey (2012)	30	Artmann (2014b)	28	Blecic et al. (2014)	10	Albino et al. (2015)	260	Farmani et al. (2012)	10	Li et al. (2017)	0
Barbour et al. (2016)	1	Barbosa et al. (2012)	135	Broto et al. (2012)	70	Angelidou (2014)	125	Grekousis et al. (2019)	0	Liu (2012)	30
Belanche et al. (2016)	34	Beck et al. (2013)	7	Chen and Chen (2019)	127	Batty et al. (2012)	372	Haapio (2012)	88	Moore et al. (2013)	56
Biddulph (2011)	29	Berta et al. (2016)	5	Chester et al. (2012)	33	Betz et al. (2016)	6	Hale and Sadler (2012)	12	Newton and Glackin (2014)	19
Blessi et al. (2012)	17	Bonafoni et al. (2017)	10	Chrysoulakis et al. (2013)	53	Bibri and Krogstie (2017)	58	Herrschel (2013)	26	Pojani and Stead (2015)	40
Bulkeley et al. (2016)	14	Breuste et al. (2013)	15	Conke and Ferreira (2015)	23	Caragliu et al. (2011)	576	Jansson (2013)	60	Thomson and Newman (2018)	4
Codecasa and Ponzini (2011)	16	Bridges (2016)	2	Cui et al. (2019)	0	Crivello (2014)	17	Jim (2013)	43	Van Timmeren et al. (2012)	6
Couch et al. (2011)	67	Chelleri et al. (2016)	2	Dijst et al. (2018)	4	Falco et al. (2018)	0	Kaur and Garg (2019)	1	Webb et al. (2018)	11
Cuthill (2010)	91	D’Al.isa et al. (2012)	32	García-Guaita et al. (2018)	1	Falco et al. (2019)	0	Rosa (2014)	43		
Deakin (2012)	11	Dierkes et al. (2015)	19	Goldstein et al. (2013)	42	Ejaz et al. (2017)	54	Rosa et al. (2017)	9		
Degen and Garcia (2012)	79	Francesch-Huidobro (2015)	8	Gonzalez et al. (2013)	47	Garau and Pavan (2018)	28	Leigh and Hoelzel (2012)	21		
Dempsey et al. (2011)	275	Gaitani et al. (2014)	14	Huang et al. (2018)	0	Garau et al. (2016)	7	Lombardi et al. (2011)	40		
Dempsey et al. (2012)	76	Girard (2013)	26	Inostroza (2014)	24	Gazzola et al. (2019)	0	MacLeo d (2013)	37		
Dixon et al. (2011)	22	Grêt-Regamey et al. (2013)	48	Kennedy et al. (2011)	258	Gharaibeh et al. (2017)	32	Marsal-Llacuna and López-Ibáñez (2014)	8		
Ergas (2010)	34	Guzmán et al. (2017)	20	Kılkış (2017)	0	Goodspeed (2014)	39	Mateo and Cunat (2016)	3		
Eriksson (2010)	28	Kaika (2017)	32	Lehmann (2011)	28	Ibrahim et al. (2018)	6	Mavrakis et al. (2015)	11		
Frantál et al. (2015)	28	Klopp and Petretta (2017)	22	Liang and Zhang (2012)	38	Lombardi et al. (2012)	158	Morimoto (2011)	10		
González et al. (2013)	166	Lapenna and Toccafondi (2017)	0	Lin et al. (2014)	27	Macke et al. (2018)	8	Mörtberg et al. (2013)	17		
Gray and Porter (2015)	19	Li et al. (2017)	17	Lund et al. (2015)	45	Manitiu and Pedrini (2016)	2	Oh et al. (2011)	5		
Guimarães (2017)	2	Lu et al. (2016)	14	Meijer (2011)	18	March and Ribera-Fumaz (2016)	32	Peng et al. (2015)	32		
Güzey (2016)	11	Marlow et al. (2013)	126	Mostafavi et al. (2014)	10	Marsal-Llacuna et al. (2015)	61	Pili et al. (2017)	51		
Haas and Locke (2012)	0	McCormick et al. (2013)	144	Mostafavi et al. (2014a)	7	Martin et al. (2018)	11	Rogers et al. (2012)	34		
Hodkinson (2011)	23	Medved (2016)	9	Niemi et al. (2012)	54	Martin et al. (2019)	0	Scott (2007)	31		
Howley et al. (2009)	73	Nevens et al. (2013)	191	Pearson et al. (2010)	74	Mosannenzadeh et al. (2017b)	15	Shi et al. (2012)	43		
Huston et al. (2015)	16	Newell et al. (2013)	41	Pincetl et al. (2012)	98	Palma Lampreia dos Santos (2016)	11	Song (2005)	55		
Jung et al. (2015)	16	Perales-Momparler et al. (2015)	13	Rosado et al. (2014)	54	Pinna et al. (2017)	10	Strazzera (2010)	13		
Keresztely and Scott (2012)	11	Pupphachai and Zuidema (2017)	13	Rosado et al. (2016)	12	Roche (2014)	36	Tyler et al. (2013)	25		
Kort and Klijn (2013)	12	Radulescu et al. (2016)	8	Shahrokni et al. (2015a)	7	Shahrokni et al. (2015)	14	Yigitcanlar and Lee (2014)	60		
Kriznik (2018)	2	Romero-Lankao et al. (2014)	24	Singh et al. (2011)	54	Shen et al. (2018	1	Zitti et al. (2015)	58		
Larco (2016)	20	Sharma et al. (2010)	26	Voskamp et al. (2018)	8	Shin et al. (2015)	19				
Lee et al. (2014)	12	Simon et al. (2015)	19	Wachsmuth (2012)	46	Soyinka et al. (2016)	3				
Lees and Melhuish (2012)	12	Stredova et al. (2015)	7	Walker and Beck (2012)	14	Steenbruggen et al. (2015)	42				
Lim et al. (2013)	16	Tran (2016)	12	Xia et al. (2018)	3	Tranos and Gertner (2012)	48				
Lugosi, et al. (2010)	17	Uyarra and Gee (2013)	27	Yang et al. (2012)	19	Winters (2011)	97				
Malleson and Heppenstall (2013)	12	Van de Meene et al. (2011)	71	Yang et al. (2014)	24	Yigitcanlar (2015)	21				
Martí-Costa and Miquel (2011)	23	Wei et al. (2015)	36	Zhang et al. (2011)	56	Yigitcanlar et al. (2019)	0				
McGuirk et al. (2016)	13	Willuweit and OSullivan (2013)	39	Zhang et al. (2014)	22	Zanella et al. (2014)	1065				
Meerkerk (2013)	25	Yang and Wang (2017)	7	Zhang et al. (2018)	1	Zhang et al. (2019)	0				
Mosannenzadeh et al. (2017)	9	Yigitcanlar and Teriman (2015)	46								
Obeng-Odoom (2014)	14	Yim et al. (2015)	3								
Pares et al. (2014)	12	Yin et al. (2014)	56								
Parés et al. (2012)	20	Yue et al. (2014)	26								
Park (2014)	2	Zhang et al. (2016)	3								
Rhodes and Russo (2013)	20	Zhao (2010)	121								
Sasaki (2010)	42	Ziervogel et al. (2016)	18								
Schuetze and Chelleri (2016)	13										
Shao and Liu (2018)	1										
Susilo et al. (2012)	39										
Tasan-Kok (2010)	25										
Tulumello (2016)	10										
Ulldemolins (2014)	22										
Uysal (2012)	23										
Van den Berg (2013)	22										
Vento (2017)	8										
While et al. (2010)	172										
Winston (2010)	43										
Zebracki and Smulders (2012)	1										
Zhong (2016)	10										

**Table A2:** Average citations and total link strength of authors per cluster based on bibliographic coupling analysis.

CLUSTER 1 (17 items)			CLUSTER 2 (14 items)			CLUSTER 3 (13 items)			CLUSTER 4 (5 items)		
Authors	Total Link Strength	Avg. Citations	Authors	Total Link Strength	Avg. Citations	Authors	Total Link Strength	Avg. Citations	Authors	Total Link Strength	Avg. Citations
Angelidou Margarita	125.38	62.50	Artmann Martina	10.00	24.00	Chester Mikhail	129.80	28.50	Davoudi Simin	89.33	7.00
Bisello Adriano	142.55	12.00	Carlucci Margherita	121.00	54.50	Chrusoulakis Nektarios	167.62	50.00	Moglia Mangus	142.00	68.50
Brandt Nils	103.81	10.5	Chelleri Lorenzo	21.75	7.50	Farzinmoghadam Mohamad	133.47	8.50	Newman Peter	292.25	6.33
De Facto Stefano	123.38	0.00	La Rosa Daniele	10.00	26.00	Gonzalez Ainhoa	167.62	50.00	Newton Peter	142.17	15.00
Evans James	147.39	5.50	Li Feng	57.00	10.00	Liu Gengyuan	98.75	29.50	Thomson Giles	292.25	6.33
Garau Chiara	93.90	15.00	Lombardi D. Rachel	136.50	37.00	Lopes Myriam	167.62	50.00			
Karvonen Andrew	147.39	5.50	Marti-Costa Marc	93.50	18.33	Lu Weisheng	43.00	2.00			
Lazarevic David	103.81	10.50	Mcguirk Pauline M.	6.00	13.50	Mostafavi Nariman	133.47	8.50			
Marsal-Llacuna Maria Luis	3.00	34.50	Pares Marc	90.50	16.00	Pincetl Stephanie	149.17	51.67			
Masala Francesca	84.12	19.00	Porter Libby	87.60	29.50	Rosado Leonardo	16.63	33.00			
Mosannenzadeh Farnaz	142.25	12.00	Rogers Chris D.F.	137.17	28.00	Spano Donatella	107.13	31.50			
Nijkamp Peter	162.33	309.0	Salvati Luca	121.00	54.50	Yang Dewei	39.10	21.50			
Pinna Francesco	84.12	19.00	Wang Rusong	45.00	36.50	Zhang Yan	105.35	27.00			
Shahrokni Hossein	103.81	10.50	Zhang Xiaoling	64.00	17.33						
Tranos Emmanuil	166.71	45.00									
Vettorato Daniele	142.55	12.00									
Yigitcanlar Tan	45.11	27.00									

**Table A3:** References from obtained results as appear first in the text

References
Dempsey, N.; Bramley, G.; Power, S.; Brown, C. The Social Dimension of Sustainable Development: Defining Urban Social Sustainability. *Sustain. Dev.* 2011, *19*, 289–300. doi:10.1002/sd.417.
While, A.; Jonas, A.E.G.; Gibbs, D. From Sustainable Development to Carbon Control: Eco-state. Restructuring and the Politics of Urban and Regional Development. *Trans. Inst. Br. Geogr.* 2010, *35*, 76–93. doi:10.1111/j.1475-5661.2009.00362.x.
González, A.; Donnelly, A.; Jones, M.; Chrysoulakis, N.; Lopes, M. A Decision-Support System for Sustainable Urban Metabolism in Europe. *Environ. Impact Assess. Rev.* 2013, *38*, 109–119. doi:10.1016/j.eiar.2012.06.007.
Cuthill, M. Strengthening the “social” in Sustainable Development: Developing a Conceptual Framework for ocial Sustainability in a Rapid Urban Growth Region in Australia. *Sustain. Dev.* 2010, *18*, 362–373. doi:10.1002/sd.397.
Degen, M.; García, M. The Transformation of the “Barcelona Model”: An Analysis of Culture, Urban. Regeneration and Governance. *Int. J. Urban Reg. Res.* 2012, *36*, 1022–1038. doi:10.1111/j.1468-2427.2012.01152.x.
Nevens, F.; Frantzeskaki, N.; Gorissen, L.; Loorbach, D. Urban Transition Labs: Co-Creating Transformative Action for Sustainable Cities. *J. Clean. Prod*. 2013, *50*, 111–122. doi:10.1016/j.jclepro.2012.12.001.
McCormick, K.; Anderberg, S.; Coenen, L.; Neij, L. Advancing Sustainable Urban Transformation. *J. Clean. Prod.* 2013, *50*, 1–11. doi:10.1016/j.jclepro.2013.01.003.
Barbosa, A.E.; Fernandes, J.N.; David, L.M. Key Issues for Sustainable Urban Stormwater Management. *Water Res.* 2012, *46*, 6787–6798. doi:10.1016/j.watres.2012.05.029.
Marlow, D.R.; Moglia, M.; Cook, S.; Beale, D.J. Towards Sustainable Urban Water Management: A Critical Reassessment. *Water Res.* 2013, *47*, 7150–7161. doi:10.1016/j.watres.2013.07.046.
Zhao, P. Sustainable Urban Expansion and Transportation in a Growing Megacity: Consequences of Urban. Sprawl for Mobility on the Urban Fringe of Beijing. *Habitat Int.* 2010, *34*, 236–243. doi:10.1016/j.habitatint.2009.09.008.
Kennedy, C.; Pincetl, S.; Bunje, P. The Study of Urban Metabolism and Its Applications to Urban Planning and Design. *Environ. Pollut*. 2011, *159*, 1965–1973. doi:10.1016/j.envpol.2010.10.022.
Chen, S.; Chen, B. Network Environ Perspective for Urban Metabolism and Carbon Emissions: A Case Study of Vienna, Austria. *Environ. Sci. Technol*. 2012, *46*, 4498–4506. doi:10.1021/es204662k.
Pincetl, S.; Bunje, P.; Holmes, T. An Expanded Urban Metabolism Method: Toward a Systems Approach for Assessing Urban Energy Processes and Causes. *Landsc. Urban Plan.* 2012, *107*, 193–202. doi:10.1016/j.landurbplan.2012.06.006.
Barles, S. Society, Energy and Materials: The Contribution of Urban Metabolism Studies to Sustainable Urban Development Issues. *J. Environ. Plan. Manag.* 2010, *53*, 439–455. doi:10.1080/09640561003703772.
Pearson, L.J.; Pearson, L.; Pearson, C.J. Sustainable Urban Agriculture: Stocktake and Opportunities. *Int. J. Agric. Sustain.* 2010, *8*, 7–19. doi:10.3763/ijas.2009.0468.
Zanella, A.; Bui, N.; Castellani, A.; Vangelista, L.; Zorzi, M. Internet of Things for Smart Cities. *IEEE Internet Things J.* 2014, *1*, 22–32. doi:10.1109/JIOT.2014.2306328.
Caragliu, A.; del Bo, C.; Nijkamp, P. Smart Cities in Europe. *J. Urban Technol*. 2011, *18*, 65–82. doi:10.1080/10630732.2011.601117.
Batty, M.; Axhausen, K.W.; Giannotti, F.; Pozdnoukhov, A.; Bazzani, A.; Wachowicz, M.; Ouzounis, G.; Portugali, Y. Smart Cities of the Future. *Eur. Phys. J. Spec. Top*. 2012, *214*, 481–518. doi:10.1140/epjst/e2012-01703-3.
Albino, V.; Berardi, U.; Dangelico, R.M. Smart Cities: Definitions, Dimensions, Performance, and Initiatives. *J. Urban Technol*. 2015, *22*, 1–19. doi:10.1080/10630732.2014.942092.
Lombardi, P.; Giordano, S.; Farouh, H.; Yousef, W. Modelling the Smart City Performance. *Innovation* 2012, *25*, 137–149. doi:10.1080/13511610.2012.660325.
Haapio, A. Towards Sustainable Urban Communities. *Environ. Impact Assess. Rev.* 2012, *32*, 165–169. doi:10.1016/j.eiar.2011.08.002.
Yigitcanlar, T.; Lee, S.H. Korean Ubiquitous-Eco-City: A Smart-Sustainable Urban Form or a Branding Hoax? *Technol. Forecast. Soc. Chang.* 2014, *89*, 100–114. doi:10.1016/j.techfore.2013.08.034.
Jansson, Å. Reaching for a Sustainable, Resilient Urban Future Using the Lens of Ecosystem Services. *Ecol. Econ.* 2013, *86*, 285–291. doi:10.1016/j.ecolecon.2012.06.013.
Zitti, M.; Ferrara, C.; Perini, L.; Carlucci, M.; Salvati, L. Long-Term Urban Growth and Land Use Efficiency in Southern Europe: Implications for Sustainable Land Management. *Sustainability* 2015, *7*, 3359–3385. doi:10.3390/su7033359.
Pili, S.; Grigoriadis, E.; Carlucci, M.; Clemente, M.; Salvati, L. Towards Sustainable Growth? A Multi-Criteria Assessment of (Changing) Urban Forms. *Ecol. Indic.* 2017, *76*, 71–80. doi:10.1016/j.ecolind.2017.01.008.
Haghshenas, H.; Vaziri, M. Urban Sustainable Transportation Indicators for Global Comparison. *Ecol. Indic.* 2012, *15*, 115–121. doi:10.1016/j.ecolind.2011.09.010.
Moore, J.; Kissinger, M.; Rees, W.E. An Urban Metabolism and Ecological Footprint Assessment of Metro Vancouver. *J. Environ. Manag.* 2013, *124*, 51–61. doi:10.1016/j.jenvman.2013.03.009.
Pojani, D.; Stead, D. Sustainable Urban Transport in the Developing World: Beyond Megacities. *Sustainability* 2015, *7*, 7784–7805. doi:10.3390/su7067784.
Liu, Y. Modelling Sustainable Urban Growth in a Rapidly Urbanising Region Using a Fuzzy-Constrained Cellular Automata Approach. *Int. J. Geogr. Inf. Sci.* 2012, *26*, 151–167. doi:10.1080/13658816.2011.577434.
Newton, P.; Glackin, S. Understanding Infill: Towards New Policy and Practice for Urban Regeneration in the Established Suburbs of Australia’s Cities. *Urban Policy Res.* 2014, *32*, 121–143. doi:10.1080/08111146.2013.877389.
Dempsey, N.; Brown, C.; Bramley, G. The Key to Sustainable Urban Development in UK Cities? The Influence of Density on Social Sustainability. *Prog. Plann.* 2012, *77*, 89–141. doi:10.1016/j.progress.2012.01.001.
Lees, L.; Melhuish, C. Arts-Led Regeneration in the UK: The Rhetoric and the Evidence on Urban Social Inclusion. *Eur. Urban Reg. Stud.* 2015, *22*, 242–260. doi:10.1177/0969776412467474.
Martí-Costa, M.; Pradel i Miquel, M. The Knowledge City against Urban Creativity? Artists’ Workshops and Urban Regeneration in Barcelona. *Eur. Urban Reg. Stud.* 2012, *19*, 92–108. doi:10.1177/0969776411422481.
Rius Ulldemolins, J. Culture and Authenticity in Urban Regeneration Processes: Place Branding in Central Barcelona. *Urban Stud.* 2014, *51*, 3026–3045. doi:10.1177/0042098013515762.
Yigitcanlar, T.; Teriman, S. Rethinking Sustainable Urban Development: Towards an Integrated Planning and Development Process. *Int. J. Environ. Sci. Technol.* 2015, *12*, 341–352. doi:10.1007/s13762-013-0491-x.
Romero-Lankao, P.; Gurney, K.R.; Seto, K.C.; Chester, M.; Duren, R.M.; Hughes, S.; Hutyra, L.R.; Marcotullio, P.; Baker, L.; Grimm, N.B.; et al. A Critical Knowledge Pathway to Low-Carbon, Sustainable Futures: Integrated Understanding of Urbanization, Urban Areas, and Carbon. *Earth’s Future* 2014, *2*, 515–532. doi:10.1002/2014ef000258.
Lu, Y.; Geng, Y.; Qian, Y.; Han, W.; McDowall, W.; Bleischwitz, R. Changes of Human Time and Land Use Pattern in One Mega City’s Urban Metabolism: A Multi-Scale Integrated Analysis of Shanghai. *J. Clean. Prod.* 2016, *133*, 391–401. doi:10.1016/j.jclepro.2016.05.174.
Chelleri, L.; Kua, H.W.; Sánchez, J.P.R.; Md Nahiduzzaman, K.; Thondhlana, G. Are People Responsive to a More Sustainable, Decentralized, and User-Driven Management of Urban Metabolism? *Sustainability* 2016, *8*, 1–12. doi:10.3390/su8030275.
Mostafavi, N.; Farzinmoghadam, M.; Hoque, S.; Weil, B. Integrated Urban Metabolism Analysis Tool (IUMAT). *Urban Policy Res*. *Taylor Fr.* 2014, 53–69. doi:10.1080/08111146.2013.826578.
Goldstein, B.; Birkved, M.; Quitzau, M.B.; Hauschild, M. Quantification of Urban Metabolism through Coupling with the Life Cycle Assessment Framework: Concept Development and Case Study. *Environ. Res. Lett.* 2013, *8*. doi:10.1088/1748-9326/8/3/035024.
Zhan, Y.; Lu, W.; Wing-Yan Tam, V.; Feng, Y. From urban metabolism to industrial ecosystem metabolism: A study of construction in Shanghai from 2004 to 2014. *J. Clean. Prod.* 2018, *202*, 428–438. doi:10.1016/j.jclepro.2018.08.054.
Broto, V.C.; Allen, A.; Rapoport, E. Interdisciplinary Perspectives on Urban Metabolism. *J. Ind. Ecol.* 2012, *16*, 851–861. doi:10.1111/j.1530-9290.2012.00556.x.
Bibri, S.E.; Krogstie, J. Smart Sustainable Cities of the Future: An Extensive Interdisciplinary Literature Review. *Sustain. Cities Soc.* 2017, *31*, 183–212. doi:10.1016/j.scs.2017.02.016.
Ahvenniemi, H.; Huovila, A.; Pinto-Seppä, I.; Airaksinen, M. What Are the Differences between Sustainable nd Smart Cities? *Cities* 2017, *60*, 234–245. doi:10.1016/j.cities.2016.09.009.
Yigitcanlar, T.; Kamruzzaman, M.; Foth, M.; Sabatini-Marques, J.; da Costa, E.; Ioppolo, G. Can Cities Become Smart without Being Sustainable? A Systematic Review of the Literature. *Sustain. Cities Soc.* 2019, *45*, 348–365. doi:10.1016/j.scs.2018.11.033.
Macke, J.; Casagrande, R.M.; Sarate, J.A.R.; Silva, K.A. Smart City and Quality of Life: Citizens’ Perception in a Brazilian Case Study. *J. Clean. Prod.* 2018, *182*, 717–726. doi:10.1016/j.jclepro.2018.02.078.
Mörtberg, U.; Haas, J.; Zetterberg, A.; Franklin, J.P.; Jonsson, D.; Deal, B. Urban Ecosystems and Sustainable Urban Development-Analysing and Assessing Interacting Systems in the Stockholm Region. *Urban Ecosyst.* 2013, *16*, 763–782. doi:10.1007/s11252-012-0270-3.
Lombardi, D.R.; Porter, L.; Barber, A.; Rogers, C.D.F. Conceptualising Sustainability in UK Urban Regeneration: A Discursive Formation. *Urban Stud.* 2011, *48*, 273–296. doi:10.1177/0042098009360690.
MacLeod, G. New Urbanism/Smart Growth in the Scottish Highlands: Mobile Policies and Post-Politics in Local Development Planning. *Urban Stud.* 2013, *50*, 2196–2221. doi:10.1177/0042098013491164.
Webb, R.; Bai, X.; Smith, M.S.; Costanza, R.; Griggs, D.; Moglia, M.; Neuman, M.; Newman, P.; Newton, P.; Norman, B.; et al. Sustainable Urban Systems: Co-Design and Framing for Transformation. *Ambio* 2018, *47*, 57–77. doi:10.1007/s13280-017-0934-6.
Thomson, G.; Newman, P. Urban Fabrics and Urban Metabolism- from Sustainable to Regenerative Cities. *Resour. Conserv. Recycl.* 2018, *132*, 218–229. doi:10.1016/j.resconrec.2017.01.010.
Davoudi, S.; Sturzaker, J. Urban Form, Policy Packaging and Sustainable Urban Metabolism. *Resour. Conserv. Recycl.* 2017, *120*, 55–64. doi:10.1016/j.resconrec.2017.01.011.
van Timmeren, A.; Zwetsloot, J.; Brezet, H.; Silvester, S. Sustainable Urban Regeneration Based on Energy Balance. *Sustainability* 2012, *4*, 1488–1509. doi:10.3390/su4071488.
